# Metabolic Disturbances Associated with In-Hospital Complication and Mortality in Different Types of Pneumonia

**DOI:** 10.3390/jcm13247832

**Published:** 2024-12-22

**Authors:** Iulia Făgărășan, Adriana Rusu, Horațiu Comșa, Maria Cristea, Nicoleta-Ștefania Motoc, Ciprian Cristea, Corina Eugenia Budin, Ruxandra-Mioara Râjnoveanu, Doina Adina Todea

**Affiliations:** 1 Department of Pneumology, “Iuliu Hațieganu” University of Medicine and Pharmacy, 400332 Cluj-Napoca, Romania; motoc_nicoleta@yahoo.com (N.-Ș.M.); dtodea@umfcluj.ro (D.A.T.); 2 Department of Diabetes and Nutrition Diseases, “Iuliu Hațieganu” University of Medicine and Pharmacy, 400006 Cluj-Napoca, Romania; adriana.rusu@umfcluj.ro; 3 Department of Cardiology, Clinical Rehabilitation Hospital, “Iuliu Hațieganu” University of Medicine and Pharmacy, 400012 Cluj-Napoca, Romania; dh.comsa@gmail.com; 4 Faculty of Electrical Engineering, Technical University of Cluj-Napoca, 26-28 G. Barițiu Street, 400027 Cluj-Napoca, Romania; maria.cristea@enm.utcluj.ro (M.C.); ciprian.cristea@emd.utcluj.ro (C.C.); 5 Department of Pathophysiology, George Emil Palade University of Medicine, Pharmacy, Science, and Technology of Târgu Mureș, 450142 Târgu Mureș, Romania; corina.budin@umfst.ro; 6 Department of Palliative Medicine, “Iuliu Hațieganu” University of Medicine and Pharmacy, 400012 Cluj-Napoca, Romania; ruxandra.rajnoveanu@umfcluj.ro

**Keywords:** COVID-19, community-acquired pneumonia, metabolic disorders, acute kidney injury, mortality

## Abstract

**Bakground:** The mortality rate from community-acquired pneumonia (CAP) or coronavirus disease 19 (COVID-19) is high, especially in hospitalized patients. This study aimed to assess the disturbances of glucose and lipid metabolism with in-hospital complications and short-term outcomes for patients with pneumonia with different etiologies. **Methods:** This observational study comprised 398 patients divided as follows: 155 with severe acute respiratory syndrome coronavirus 2 (SARS-CoV-2) pneumonia, 129 participants with viral CAP, and 114 with bacterial pneumonia. **Results:** Fasting plasma glucose (FPG) at admission and glycemic variation during hospitalization was linked with acute kidney injury (AKI) in bacterial CAP. Compared with a value <110 mg/dL for FPG at admission, levels between 110 and 126 mg/dL are associated with mortality in both COVID-19 (OR = 3.462, 95% CI: 1.275–9.398, *p* = 0.015) and bacterial CAP participants (OR = 0.254; 95% CI: 0.069–0.935, *p* = 0.039), while a value ≥126 mg/dL was linked with mortality only in patients with SARS-CoV-2 (OR = 3.577, 95% CI: 1.166–10.976, *p* = 0.026). No relation between lipid biomarkers and complications or in-hospital outcomes was observed in all three participant groups. **Conclusions:** Patients with bacterial CAP are more prone to developing AKI due to increased FBG at admission and glycemic variations during hospitalization, while elevated FBG values at admission are associated with mortality in both COVID-19 and bacterial CAP.

## 1. Introduction

Community-acquired pneumonia (CAP) is associated with an increased rate of morbidity and mortality, especially in elderly patients or those with chronic diseases [1]. A series of risk factors for CAP occurrence were proposed [2], but the latest studies reported a connection between inter-human interactions and asymptomatic carriers with bacterial CAP development, in particular with *Streptococcus Pneumoniae* [3,4]. Besides the bacterial etiology, respiratory viruses are a frequent cause of pneumonia, being more often detected when they are methodically tested [5]. From 2019, a new cause of viral pneumonia has been identified, given by severe acute respiratory syndrome coronavirus 2 (SARS-CoV-2). This leads to interstitial and bilateral pneumonia, with varying degrees of severity and complication, including respiratory failure and acute respiratory distress syndrome (ARDS); acute kidney, liver, or cardiac injury; disseminated intravascular coagulation; or mortality [6,7].

Patients with underlying conditions are prone to infection and developing severe forms of disease [8]. A chronic inflammatory state is associated with underlying illnesses, such as obesity, diabetes mellitus (DM), cardiovascular, and chronic respiratory diseases, and is linked with high circulating levels of pro-inflammatory cytokines during SARS-CoV-2 infection, known as cytokine release syndrome or cytokine storm [9]. A hyperinflammatory state may be associated with an imbalance in glucose metabolism. Regardless of the presence or absence of DM, hyperglycemia may occur in patients with pneumonia [10] and is known to be a risk factor for mortality in CAP [11]. A large body of scientific evidence reported a link between hyperglycemia and morbidity and mortality in patients who are critically ill [12,13], including in patients with COVID-19 [14].

In addition to an accentuated inflammatory status, lipid metabolism also plays an important role in the progression of an infectious disease [15]. Cholesterol is a component of the viral membrane envelope, and the available evidence suggests it plays an important role in the cell entrance of the SARS-CoV-2 virus [16]. The continuous interaction between serum lipids and those found in cell membranes can lead to a more severe form of the disease [17,18].

In this study, we aim to investigate whether the association of fasting plasma glucose (FPG) and/or dyslipidemia with in-hospital complications and short-term outcomes for patients with pneumonia is influenced by the etiology of pneumonia in order to improve the current clinical practice and better understand the association between metabolic disturbances and the risk of complications and death.

## 2. Materials and Methods

### 2.1. Study Design and Population

This retrospective study investigated a cohort of 398 participants admitted to the “Leon Daniello” Pulmonology Hospital of Cluj-Napoca, Romania. The patients with COVID-19 were those admitted from 1 January 2021 to 30 June 2021, while data for participants with viral and bacterial pneumonia were collected from those admitted prior to the COVID-19 global pandemic—from 1 January 2017 to 31 December 2018. The study complied with the principles of the Declaration of Helsinki and was approved by the Ethics Committee for Human Research (approval No. 182/21 July 2023 and No. 10/31 January 2024), and all the patients provided informed consent at admission.

The inclusion criteria of patients in the study included the following: (1) age >18 years old; (2) a laboratory-confirmed diagnosis of the SARS-CoV2 infection by a real-time polymerase chain reaction (RT-PCR) of a nasopharyngeal swab for patients with COVID-19; (3) clinical manifestation (such as fever, cough, expectoration with or without purulent sputum, chest pain, and shortness of breath) and imaging investigations suggestive of viral and bacterial pneumonia; (4) patients who followed chronic treatment at home before admission, according to medical recommendations; (5) glycemic control and diabetes consultation in the last 6 months.

Exclusion criteria comprised patients who had the following: (1) signs of nosocomial infections; (2) those who underwent surgery within 3 months prior to infection; (3) pneumonia with atypical mycobacteria and fungal pneumonia; (4) subjects who were transferred to another hospital for medical care; (5) patients with duplicate records during the studied periods (participants admitted twice for pneumonia); or (6) patients with missing clinical, biochemical, or radiological investigation.

### 2.2. Data Collection and Definition

General information about the patients, including age, gender, body mass index (BMI), comorbidities: DM, hypertension, coronary heart disease, chronic heart failure, cerebrovascular disease, asthma, chronic obstructive pulmonary disease, symptoms at admission, arterial blood gas, and computed tomography (CT) or chest radiography imaging were recorded from the hospital electronic database. All the laboratory analyses were collected in the morning before breakfast and were performed using standardized methods in the hospital’s central laboratory. Blood sample data were collected on admission day, the minimum and maximum values during hospitalization, and at discharge for the following tests: complete blood count (CBC), CRP, lactate dehydrogenase (LDH), liver and renal function, fasting plasma glucose (FPG), and blood lipid levels, along with bacterial culture. Glycemic variation during hospitalization was calculated as the difference between the maximum and the minimum glycemic value recorded in patients’ files. Triglyceride–glucose index (TyG index) was calculated as Ln (fasting triglycerides [mg/dL] × fasting glucose [mg/dL]/2), while the triglyceride–glucose body mass index (TyG-BMI index) was the result of TyG × BMI [(weight/height)^2^]. The complications and outcomes for each patient were added. CURB-65 score was used as a prognostic score tool in predicting 30-day mortality for each patient.

SARS-CoV-2 pneumonia was diagnosed if the patients had a positive test for SARS-CoV-2 infection by the reverse transcription polymerase chain reaction and ground-glass opacities or consolidation on chest CT or pulmonary infiltrates/new patchy infiltrate on chest X-ray. The diagnostic criteria for viral pneumonia included interstitial infiltrate changes on chest radiography or a CT with at least one of the following: recent presence of sputum, cough or dyspnea, peripheral white cell counts > 10 × 10^9^/L or <4 × 10^9^/L, or temperature > 38.0 °C [19]. The diagnosis of bacterial pneumonia was made if a bacterium was identified on spontaneous sputum examination (routinely performed) or bronchoalveolar lavage (only when needed), accompanied by the presence of consolidation with air bronchogram.

At admission, the CURB-65 score [20,21] was performed to predict in-hospital mortality of both COVID-19 and community-acquired pneumonia.

We defined the clinical outcomes as the following events: clinical complication (acute kidney injury or respiratory failure), short-term outcomes (intensive care unit (ICU) admission), and death during the hospitalization. According to the KDIGO guidelines [22], acute kidney injury was defined as an increase in serum creatinine value ≥ 0.3 mg/dL (≥26.5 μmol/L) within 48 h, or an increase in serum creatinine ≥ 1.5 folds the baseline value (known value or the value of the last 7 days), or less than 0.5 mL/kg/hour in urine volume for six hours. Respiratory failure was characterized by abnormalities in arterial blood gas, with hypoxemia, hypercapnia, or both. The decision to transfer patients to the ICU was based on the modified national early warning score (Modified NEWS) [23] for patients with COVID-19 and on the American Thoracic Society (ATS) severity criteria from 2007 [24] for pneumonia of other etiologies.

### 2.3. Statistical Method

The categorical variables are presented as numbers (percentages) and compared between pneumonia groups using the chi square test of Fisher’s exact test. Continuous variables are presented as mean and standard deviation (SD) or as median and interquartile range (IQR). The differences between groups for continuous and categorical variables were tested using Mann–Whitney U test, independent samples median test, and Kruskal–Wallis one-way ANOVA. The association of variables collected with outcomes of interest was tested using univariate logistic regression adjusted for age, sex, and pre-existing comorbidities (arterial hypertension, chronic obstructive pulmonary disease, chronic kidney disease, cerebrovascular disease, coronary heart disease, heart failure, and cancer). Variables associated with any of the in-hospital complications in the univariate regression models were further included in a multivariate logistic regression model. A two-sided *p* < 0.05 was regarded as statistically significant. All analyses were performed using IBM SPSS Statistics V26.0 (IBM Corp.: Armonk, NY, USA).

## 3. Results

### 3.1. Demographic and Baseline Characteristics of the Participants

**Figure 1:**
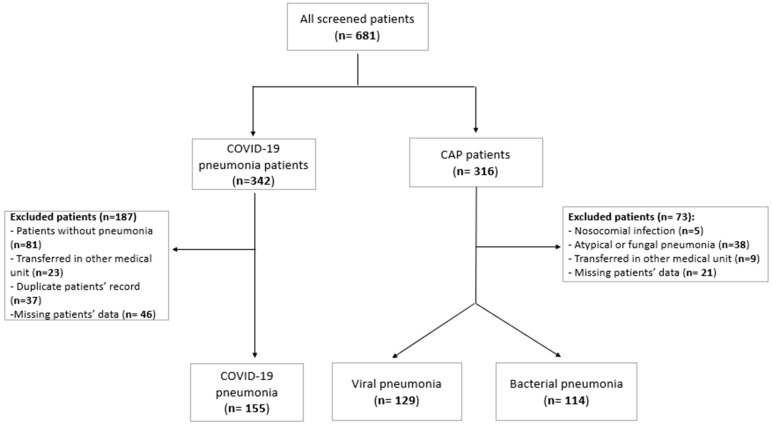
Flow chart of the participants’ selection.

During the study period, 398 patients were included in the present research: 155 (38.94%) with COVID-19 pneumonia, 129 (32.41%) with viral pneumonia, and 114 (28.64%) with bacterial pneumonia. The selection of the participants can be seen in Figure 1.

The patients’ characteristics and comorbidities, along with signs and symptoms, are summarized in Table 1.

Patients with COVID-19 were older than patients with viral or bacterial pneumonia (66.6 years vs. 62.1 years, 64.7 years, *p* = 0.02), with a higher BMI (*p* = 0.001), and had a higher prevalence of preexisting DM and hypertension (both with *p* < 0.0001) than those with bacterial or other viral pneumonia. Regarding the underlying disease in bacterial pneumonia, patients had more frequently coronary heart disease and chronic obstructive pulmonary disease (both with *p* < 0.0001) than patients with COVID-19 or other viral pneumonia. Patients with COVID-19 pneumonia had fever and cough more often than the other two groups of patients with pneumonia, both with a *p*-value < 0.05. Laboratory findings at admission that were recorded on the first day of hospitalization were compared between groups, as shown in Table 2.

**Figure 2:**
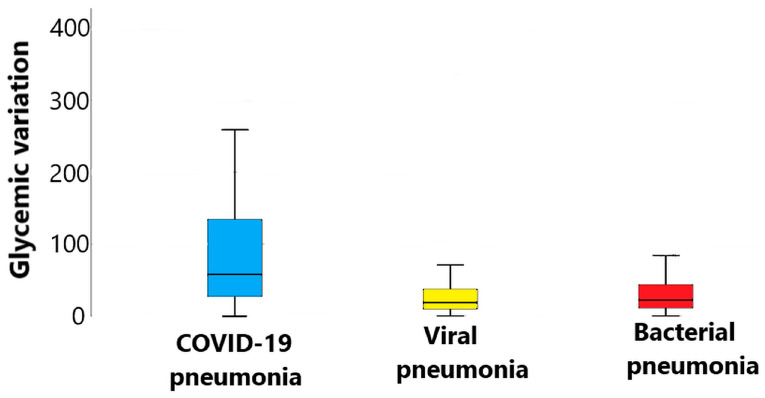
Glycemic variation between COVID-19, viral and bacterial pneumonia groups. *p* < 0.001 for the difference between COVID-19 and the other etiologies. *p* = 1.000 for the difference between viral and bacterial pneumonia.

In terms of biomarkers, higher values at admission were observed for glycemia (162.2 mg/dL, *p* = 0.001) and triglycerides (211.6 mg/dL, *p* < 0.0001) in patients with COVID-19 pneumonia than in viral or bacterial CAP groups. Triglyceride–glucose index (TyG index) and triglyceride–glucose–body mass index (TyG-BMI index) were higher in patients with SARS-CoV-2 (both with *p* = 0.001) compared to the other 2 groups. Also, glycemic variation was significantly higher in participants with COVID-19 pneumonia than in the other etiology groups (*p* < 0.001)—Figure 2.

Although the inflammatory syndrome was more pronounced at admission among patients with COVID-19 pneumonia, no statistically significant difference was observed between the studied groups (*p* = 0.18), while LDH was statistically significant in patients with SARS-CoV-2-*p* = 0.001. Also, these patients presented a more pronounced hypoxemia with a more severe ARDS compared to patients with viral or bacterial CAP—*p* = 0.001.

**Figure 3:**
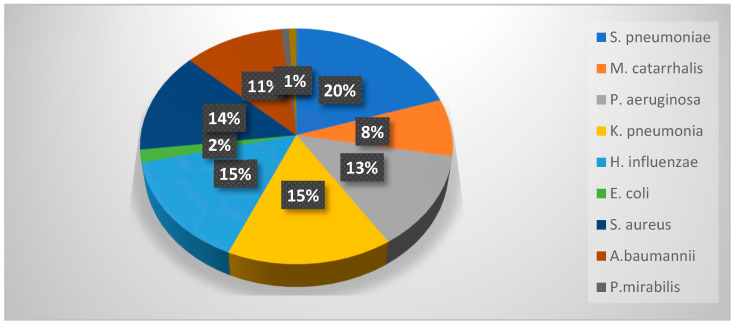
Bacterial CAP etiology; *S. pneumoniae*—Streptococcus pneumoniae; *M. catarrhalis*—Moraxella catarrhalis; *P. aeruginosa*—Pseudomonas aeruginosa; *K. pneumoniae*—Klebsiella pneumoniae; *E. coli*—Escherichia coli; *S. aureus*—Staphylococcus aureus; *A. baumannii*—Acinetobacter baumannii; *P. mirabilis*—Proteus mirabilis.

Regarding the bacterial species observed in patients with bacterial pneumonia, they are presented in Figure 3.

### 3.2. Complications and Outcomes

In terms of complication and outcomes, Table 3 summarizes the most important results.

In patients with COVID-19 pneumonia, participants needed oxygen therapy more frequently—60.6% vs. 20.7% vs. 25.5%, *p* < 0.0001; had a higher rate of acute respiratory failure—51.5% vs. 28.6% vs. 24.2%, *p* < 0.001; and acute renal failure—37.3% vs. 24.4% vs. 23.9%, *p* = 0.021, than patients with viral or bacterial pneumonia CAP. Also, a higher need for ICU admission was seen in the COVID-19 pneumonia group when compared to the other two groups—20.0% vs. 0.8% vs. 6.1%, *p* < 0.0001. Mortality (27.1% vs. 7.8% vs. 27.2%, *p* < 0.0001) and the length of hospital stay (12.8 days vs. 11.0 days vs. 13.3 days, *p* = 0.01—Table 1) were similar in the COVID-19 and bacterial pneumonia groups but significantly higher when compared to pneumonia of other viral etiologies.

### 3.3. The Odds Ratio of Complication and Risk of Mortality in Patients with Different Types of Pneumonia

Table 4 presents the adjusted odds ratio of in-hospital complications and clinical outcomes of patients with different etiologies of pneumonia stratified by different values for FPG.

In patients with COVID-19, the FPG levels between 110–126 mg/dL and ≥126 mg/dL were associated with a significantly higher mortality (OR = 3.462, 95% CI: 1.275–9.398, *p* = 0.015; OR = 3.577, 95% CI: 1.166–10.976, *p* = 0.026) than FPG < 110 mg/dL. Also, in the bacterial pneumonia group, FPG between 110 and 126 mg/dL was associated with acute kidney injury (OR = 0.240, 95% CI: 0.062–0.923, *p* = 0.038) and mortality (OR = 0.254, 95% CI: 0.069–0.935, *p* = 0.039) compared to other FPG values. No associations between FPG and in-hospital complications and outcomes were observed for other types of viral pneumonia.

Furthermore, we investigated whether glycemic variation interferes with the presence of complications and the outcomes of this study; results are presented in Table 5. The only complication associated with glycemic fluctuation was acute kidney disease in the bacterial pneumonia group—OR = 1.036, 95% CI: 1.015–1.058, *p* = 0.001.

Table 6 presents the impact of lipid biomarkers on complications and outcomes in all three patients with pneumonia. No association of either cholesterol or triglyceride levels was observed with in-hospital complications and outcomes in any of the pneumonia groups.

As for the inflammatory syndrome expressed by CRP, a high value on admission was associated with higher mortality in the bacterial pneumonia group (OR = 1.039, 95% CI: 1.019–1.060, *p* < 0.0001)—Table 7.

Variables that were associated with any of the in-hospital complications in the univariate regression models (i.e., fasting plasma glucose levels at admission, glycemia variation during the hospitalization, and CRP at admission) were further included in a multivariate regression model (Table 8). In this model, FPG at admission remained associated with a higher mortality during COVID-19 infection (OR = 1.750, 95% CI: 1.113–2.754). Glycemia variation was associated with a higher risk of acute kidney injury in bacterial infection, while CRP levels at admission were associated with higher mortality in bacterial infection. As in the univariate models, none of the biomarkers assessed were associated with any studied outcomes during the viral infection.

## 4. Discussion

The present study, based on our information, is the first to show that glucose levels on admission and glycemic variation during hospitalization were associated with acute kidney injury in patients with bacterial CAP, irrespective of their glycemic status before CAP. While the FPG on admission shows the acute stress response (“fight or flight”), glycemic variation—as a part of glucose homoeostasis—is expressed by glycemic excursion, leading to the risk of hyperglycemia or hypoglycemia [25]. In DM patients with acute myocardial infarction, Gao et al. [26] have shown that stress hyperglycemia ratio on admission (defined by admission blood glucose divided by the glycated hemoglobin-derived estimated average glucose) is associated with AKI and in-hospital morbidity and mortality. The pathogenetic mechanism linking AKI and acute hyperglycemia during pneumonia could involve inflammatory cytokines. Preclinical studies showed that tumor necrosis factor alpha (TNF-α) and interleukin-6 (IL-6) can determine down-regulation of GLUT-4 glucose transporters in the peripheral cells and impair post-receptor insulin signaling [27]. Other potential mechanisms involved in the alteration of renal function are the maintenance and worsening of the inflammatory status and oxidative stress by acute hyperglycemia [28], which are more pronounced in patients with bacterial CAP. Furthermore, an excessive inflammatory response may drive AKI through a direct detrimental activity of circulating cytokines and chemokines that promote tubular epithelial cell apoptosis [29]. Several previous investigations have proved that in critical condition, the occurrence of glycemic variations outside the physiological limits (70 to 140 mg/dL) is associated with increased mortality, irrespective of the presence or absence of diabetes [14,30].

For the patients who experience infection or sepsis, the activation of stress is responsible for the disorder of the glucose metabolism, especially hyperglycemia and glycemic variation [31]. In case of infection, the release of inflammatory mediators and cytokines leads to a higher hepatic gluconeogenesis and insulin resistance [32]. Studies published so far suggested that several factors participate in the occurrence of AKI in patients with sepsis: inflammation, microvascular flow dysfunction at the peritubular and glomerular levels, increased oxidative stress at the tubular epithelial cell level, with consecutive down-regulation of cellular metabolism and the interruption of the cell cycle [33]. Data available in the literature showed that glucose fluctuations (acute and chronic) can further increase the oxidative stress, mitochondrial damage, and inflammation and has been associated with increased mortality in the patients in the ICU [34,35,36]. Furthermore, animal models and studies performed in patients with AKI showed that glucose variability accelerates kidney injury and is a prognostic marker for mortality in patients with AKI [35], supporting our findings on the relationship between glucose variability and AKI.

Considering that in the bacterial pneumonia group, the patients presented more frequently with coronary heart disease and chronic obstructive pulmonary disease compared with the other two groups, the increase of AKI can also be attributed to polypharmacy (such as antibiotics or cardiovascular medication), results that are in line with a report published by Chao et al. [37]. It was previously proved that impaired renal perfusion along with reduction of the glomerular filtration rate, nephrotoxicity through interaction with other drugs, and side effects of the medication could precipitate AKI in patients with chronic cardiovascular treatment [37]. Non-steroidal anti-inflammatory drugs, more frequently used with increasing age, are another class of concern with a negative effect on renal function [38,39]. Although we did not assess the use before admission of this class of drugs, we cannot exclude a mild pre-existing renal impairment associated with it, which may have precipitated the development of AKI in the context of glucose variations, inflammation, and oxidative stress.

The present findings have demonstrated that FPG on admission is associated with in-hospital mortality in COVID-19 pneumonia and bacterial CAP. In patients with COVID-19, elevation of glycemia was seen even in patients without diabetes, and it was linked with a longer hospital stay, need for ICU, and higher risk of mortality [40]. Also, hyperglycemia on admission was associated with a poor prognosis in patients with COVID-19, as the PISA COVID-19 study has reported previously [41]. In Yang’s study [42], the multivariable Cox regression analyses showed that an FPG value between 126–200 mg/dL and ≥200 mg/dL is an independent predictor for 28-day mortality in patients with SARS-CoV-2 infection. Considering that COVID-19 infection was associated with the occurrence of hyperglycemia and new-onset diabetes [43], it is believed that the virus directly affects β-pancreatic cells due to increased inflammation with secondary reduction in insulin secretion, but also with insulin resistance probably due to higher levels of counterregulatory hormones and cytokines [44,45], highlighted in the present study by increasing the TyG and TyG-BMI index. Also, as patients were quarantined, they had a period of reduced physical activity, with reduced insulin sensitivity.

Regarding bacterial CAP, a value > 110 mg/dL was associated with mortality in the present study, results that are in line with the previous literature [11]. Several underlying mechanisms responsible for the occurrence of hyperglycemia in patients who are critically ill have been described: the absolute or relative insulin deficiency and impaired glucose metabolism [46]. Also, in patients with severe illness, along with pronounced inflammation, the sympatho-adrenal system and hypothalamic–pituitary–adrenal axis are activated, which maintain insulin resistance with secondary hyperglycemia [47]. In patients with severe conditions, hyperglycemia is more toxic than in patients who are non-critically ill, where the cells have a protective role by down-regulating glucose transporters [48]. This phenomenon is supported by the accentuated toxic side effect of glycolysis along with oxidative phosphorylation [49].

The results presented above suggest that hyperglycemia at presentation and glycemic variations during hospitalization have a crucial role in AKI development and mortality in patients with severe pneumonia. Although the association found is weak (ORs < 2) and may suggest the presence of other confounding factors that could explain our results and which were not included in the analyses, this association may also suggest that the infection is the driving force precipitating both the hyperglycemia and the complications associated with severe pneumonia.

CRP was the main inflammatory biomarker studied in the present research. Admission values were associated with mortality in bacterial pneumonia, results that are similar to those from prior studies [50]. A prospective study published by Menendez et al. [51] showed that the admission value for CRP is a predictor of mortality in a 30-day follow-up study in patients with CAP, after adjusting for the CURB-65 prognostic scale (AUC from 0.82 to 0.85) and prognostic severity index (PSI)-AUC from 0.80 to 0.85. Furthermore, a recent meta-analysis has revealed that CRP is a more reliable marker for mortality-AUC = 0.8, along with leukocytosis-AUC = 0.77, and procalcitonin (PCT)-AUC = 0.77 [52], although Nouvenne et al. [53] proved that CRP is a more accurate marker for predicting the diagnosis of pneumonia, while PCT is more specific for bacteremia with identification of bacterial species and differential diagnosis between bacterial vs. nonbacterial infections. A possible explanation to support the results of the present study would be the fact that patients with bacterial pneumonia have a more pronounced inflammatory storm compared to patients with COVID-19 pneumonia and viral CAP.

The present study has several limitations. The first one is represented by the single-center design and its modest sample size. However, all consecutive participants were included, thus reflecting the real-world scenario. Secondly, considering the retrospective nature of the study, we did not have measurements of inflammatory biomarkers and glycemic values before admission; thus, it cannot be concluded whether the inflammation/hyperglycemia detected during hospitalization was the consequence of the acute disease or reflected the inflammatory and glycemic state given by the underlying diseases. Thirdly, considering the lack of PCT dosing, it is plausible that some cases of pneumonia classified as being due to a viral cause were actually bacterial pneumonia or vice versa. According to the regional protocol of the hospital where the study was performed, PCT determination is not routinely tested due to financial reasons, thus limiting its routine use. Future, prospective, long-term follow-up studies are needed in order to confirm the present results.

## 5. Conclusions

Considering the importance of pneumonia on global morbidity and mortality, the present study showed for the first time that admission FPG levels and glycemic variation were linked with AKI in bacterial CAP, providing additional information to the scarce data available in the literature. This could be clinically relevant in monitoring the occurrence of possible immediate complications in patients with bacterial pneumonia and early initiation of antihyperglycemic treatment. In addition, our findings suggest that there is an association between FPG on admission day and mortality in patients with COVID-19 and bacterial pneumonia. Finding biomarkers associated with in-hospital complications or mortality can bring an individualized therapeutic decision that may reduce the poor evolution and the mortality in patients with pneumonia.

## Figures and Tables

**Table 1:** Baseline characteristics of the participants.

Variable	COVID-19 Pneumonia (*n* = 155)	Patients with Pneumonia with Viral Infection (*n* = 129)	Patients with Pneumonia with Bacterial Infection (*n* = 114)	*p* Value
Demographic characteristics at admission-no. [Q1;Q3]				
Age, years	66.6 [33.0; 93.0]	62.2 [19.0; 92.0]	64.8 [32.0; 93.0]	0.02
Men, no. (%)	103.0 (65.6%)	79.0 (60.3%)	85.0 (73.3%)	0.086
BMI, kg/m^2^	34.3 [23.9; 49.1]	29.2 [16.5; 45.6]	33.0 [16.9; 42.7]	0.001
Systolic blood pressure, mmHg	130.0 [80.0; 200.0]	125.0 [80.0; 200.0]	129.0 [80.0; 180.0]	0.06
Diastolic blood pressure, mmHg	77.0 [50.0; 105.0]	76.0 [50.0; 100.0]	77.0 [60.0; 100.0]	0.85
Respiratory rate	20.0 [15.0; 38.0]	18.0 [15.0; 35.0]	19.0 [16.0; 34.0]	0.001
Pre-existing comorbidities, no. (%)				
Diabetes mellitus	66.0 (42.6%)	18.0 (14%)	24.0 (21.1%)	<0.0001
Hypertension	120.0 (77.4%)	58.0 (45.3%)	62.0 (54.4%)	<0.0001
Coronary heart disease	7.0 (4.5%)	20.0 (15.5%)	23.0 (20.2%)	<0.0001
Chronic heart failure	20.0 (12.9%)	21.0 (16.3%)	21.0 (18.4%)	0.451
Cerebrovascular disease	13.0 (8.4%)	10.0 (7.8%)	10.0 (8.8%)	0.958
Asthma	16.0 (10.3%)	5.0 (3.9%)	12.0 (10.5%)	0.086
Chronic obstructive pulmonary disease	14.0 (9.0%)	7.0 (5.4%)	49.0 (43.0%)	<0.0001
Sign and symptoms, no. (%)				
Fever	76.0 (49.0%)	38.0 (29.9%)	38.0 (33.3%)	0.002
Cough	106.0 (68.4%)	89.0 (70.1%)	94.0 (82.5%)	0.025
Shortness of breath	105.0 (67.7%)	75.0 (59.1%)	80.0 (70.2%)	0.151
Diarrhea	19.0 (12.3%)	2.0 (1.6%)	0	<0.0001
Fatigue	46.0 (29.8%)	44.0 (34.6%)	36.0 (31.6%)	0.671
Chest pain	18.0 (11.6%)	44.0 (34.4%)	36.0 (31.6%)	<0.0001
Onset symptoms until admission, days	5.1 [0.0–21.0]	9.7 [1.0–25.0]	11.5 [2.0–26.0]	0.001
CURB-65 score				
0	25.0 (16.1%)	58.0 (45.0%)	32.0 (28.1%)	<0.0001
1	50.0 (32.3%)	46.0 (35.7%)	49.0 (43.0%)	
2	48.0 (31.0%)	25.0 (19.4%)	25.0 (21.9%)	
3	23.0 (14.8%)	0	4.0 (3.5%)	
4	8.0 (5.2%)	0	4.0 (3.5%)	
5	1.0 (0.6%)	0	0	
Hospitalization length, days	12.8 [3.0–40.0]	11.0 [2.0–32.0]	13.3 [4.0–32.0]	0.01

BMI—body mass index.

**Table 2:** Laboratory findings and arterial blood gas at admission.

Variable	COVID-19 Pneumonia (*n* = 155)	Patients with Pneumonia with Viral Infection *(n* = 129)	Patients with Pneumonia with Bacterial Infection (*n* = 114)	*p* Value
Laboratory findings at admission-median, [Q1; Q3]				
White blood cells, ×10^3^/L	8.41 [0.68–19.98]	9.69 [3.5–24.43]	10.29 [2.26–25.02]	0.001
Hemoglobin, g/dL	13.07 [7.8–17.0]	12.72 [7.5–17.6]	12.56 [6.6–17.0]	0.08
Plateles count, ×10^3^/L	259.05 [32.8–825]	310.13 [84–747.0]	274.66 [15.7–806.0]	0.001
ASAT, U/L	48.76 [14–381]	36.14 [10–212]	31.64 [10–98]	0.001
ALAT, U/L	45.20 [7–290]	42.71 [10–239]	31.46 [10.129]	0.009
Creatinine, mg/dL	1.33 [0.66–5.5]	1.11 [0.6–3.6]	1.28 [0.67–5.6]	0.44
BUN, mg/dL	30.20 [6.8–102.6]	18.08 [1.6–143.7]	25.76 [4.1–153.5]	0.001
Fasting plasma glucose, mg/dL	162.23 [69.0–500.0]	119.03 [62.0–367.0]	127.13 [75.0–297.0]	0.001
Total cholesterol, mg/dL	153.16 [70.0–284.0]	177.61 [80.0–364.0]	175.33 [85.0–312.0]	0.001
Triglycerides, mg/dL	211.62 [47.0–808.0]	137.14 [40.0–393.0]	125.40 [35.0–370.0]	<0.0001
TyG	5.11 [4.30–6.13]	4.76 [4.08–5.44]	4.74 [4.01–5.42]	0.001
TyG-BMI index	177.98 [121.12–287.64]	143.90 [76.05–238.53]	160.10 [74.92–202.83]	0.001
LDL-C, mg/dL	139.36 [25.9–239.8]	174.16 [123.8–251.7]	160.69 [126.2–219.3]	0.01
HDL-C, mg/dL	38.51 [14.2–51.9]	42.90 [20.0–81.8]	45.24 [27.0–71.5]	0.08
C-reactive protein, mg/L	65.36 [3.10–101.7]	58.39 [0.5–369.0]	56.32 [0.5–137.6]	0.18
Lactate dehydrogenase, U/L	664.03 [178–2528]	421.61 [203–1207]	391.81 [162–1672]	0.001
Arterial blood gas at admission				
pH	7.43 [7.05–7.59]	7.39 [7.2–7.59]	7.38 [7.23–7.5]	0.001
PaCO_2_, mmHg	33.61 [18.0–60.0]	36.15 [21.0–67.9]	39.01 [21.0–88.0]	0.001
PaO_2_, mmHg	59.71 [24.8–115.0]	65.70 [46.0–83.0]	66.43 [39.4–88.9]	0.008
SaO_2_, %	86.93 [26.0–99.0]	91.58 [77.0–98.0]	88.96 [67.0–97.0]	0.04
HCO_3_, mmol/L	24.52 [12.1–35.9]	27.44 [18.8–46.0]	27.07 [13–43.0]	0.003
PaO_2_/FiO_2_	140.60 [166.0–376.19]	263.24 [213.0–290.0]	248.83 [203.0–291.1]	0.001

ASAT—aspartate aminotransferase; ALAT—alanine aminotransferase; BUN—blood urea nitrogen; TyG—triglyceride–glucose index; TyG-BMI index—triglyceride–glucose–body mass index; LDL-C—low-density lipoprotein cholesterol; HDL-C—high-density lipoprotein cholesterol; PaO_2_—partial pressure of oxygen; PaCO_2_—partial pressure of carbon dioxide; PaO_2_/FiO_2_—the ratio of partial pressure of oxygen in arterial blood to the fraction of inspiratory oxygen concentration.

**Table 3:** Complications and clinical outcomes of the participants.

Variable	COVID-19 Pneumonia (*n* = 155)	Patients with Pneumonia with Viral Infection (*n* = 129)	Patients with Pneumonia with Bacterial Infection (*n* = 114)	*p* Value
Clinical outcome and complication, no. (%)				
Oxygen				<0.0001
- No need	26.0 (16.8%)	90.0 (74.4%)	60.0 (54.5%)	
- Oxygen therapy	94.0 (60.6%)	25.0 (20.7%)	28.0 (25.5%)	
- NIV	17.0 (11.0%)	6.0 (5.0%)	22.0 (20.0%)	
- MV	18.0 (11.6%)	0	0	
Acute respiratory failure	51.0 (51.5%)	12.0 (28.6%)	15.0 (24.2%)	0.001
Acute renal failure	57.0 (37.3%)	31.0 (24.4%)	27.0 (23.9%)	0.021
Need of ICU, no (%)	31.0 (20.0%)	1.0 (0.8%)	7.0 (6.1%)	<0.0001
Death, no (%)	42.0 (27.1%)	10.0 (7.8%)	31.0 (27.2%)	<0.0001

NIV—non-invasive ventilation; MV—mechanical ventilation; ICU—intensive care unit.

**Table 4:** Adjusted odds ratios of in-hospital complications and outcomes in patients with pneumonia by etiology and fasting plasma glucose level at admission.

Complication and Outcome	COVID-19		Viral Infection		Bacterial Infection	
	Adjusted OR (95% CI)	*p*-Value	Adjusted OR (95% CI)	*p*-Value	Adjusted OR (95% CI)	*p*-Value
Acute kidney injury						
110–126 mg/dL vs. <110 mg/dL	0.368 (0.129–1.053)	0.062	0.949 (0.302–2.981)	0.928	0.240 (0.062–0.923)	0.038
≥126 mg/dL vs. <110 mg/dL	2.363 (0.825; 6.766)	0.109	0.966 (0.253; 3.694)	0.960	0.341 (0.078; 1.497)	0.154
Respiratory failure						
110–126 mg/dL vs. <110 mg/dL	0.876 (0.272–2.827)	0.825	0.195 (0.016–2.386)	0.201	2.285 (0.233–22.40)	0.478
≥126 mg/dL vs. <110 mg/dL	0.654 (0.195; 2.194)	0.491	0.037 (0.001; 2.278)	0.117	2.242 (0.307; 16.359)	0.426
ICU						
110–126 mg/dL vs. <110 mg/dL	2.498 (0.949–6.573)	0.064	NA		0.004 (0.000–11.60)	0.176
≥126 mg/dL vs. <110 mg/dL	1.374 (0.420; 4.494)	0.599	NA		1.128 (0.032; 39.315)	0.947
Death						
110–126 mg/dL vs. <110 mg/dL	3.462 (1.275–9.398)	0.015	1.842 (0.124–27.46)	0.658	0.254 (0.069–0.935)	0.039
≥126 mg/dL vs. <110 mg/dL	3.577 (1.166; 10.976)	0.026	4.488 (0.201; 100.33)	0.344	0.946 (0.259; 3.447)	0.933

ICU—intensive care unit. Parameters included in the adjustment include the following: age, sex, and pre-existing comorbidities (arterial hypertension, chronic obstructive pulmonary disease, chronic kidney disease, cerebrovascular disease, coronary heart disease, heart failure, and cancer). NA—non applicable.

**Table 5:** Association of glycemia variation during hospitalization with in-hospital complications by pneumonia etiology.

Complication and Outcome	COVID-19		Viral Infection		Bacterial Infection	
	Adjusted OR (95% CI)	*p*-Value	Adjusted OR (95% CI)	*p*-Value	Adjusted OR (95% CI)	*p*-Value
Acute kidney injury	1.002 (0.998–1.006)	0.389	1.005 (0.986–1.024)	0.604	1.036 (1.015–1.058)	0.001
Respiratory failure	1.000 (0.995–1.005)	0.902	0.979 (0.935–1.024)	0.358	1.023 (0.996–1.052)	0.095
ICU	1.002 (0.997–1.007)	0.360	NA		1.042 (0.996–1.090)	0.074
Death	1.001 (0.996–1.005)	0.833	0.962 (0.909–1.018)	0.177	1.008 (0.993–1.024)	0.285

ICU—intensive care unit. Glycemia variation during hospitalization was calculated as the difference between the maximum and the minimum glucose level recorded in patient files. Glycemia variation was included as a continuous variable in the logistic regression model. Parameters adjusted include age, sex, and pre-existing comorbidities (arterial hypertension, chronic obstructive pulmonary disease, chronic kidney disease, cerebrovascular disease, coronary heart disease, heart failure, and cancer). NA—non applicable.

**Table 6:** Adjusted odds ratios of in-hospital complications in patients with pneumonia by etiology and cholesterol and triglyceride levels at admission.

Complication and Outcome	COVID-19		Viral Infection		Bacterial Infection	
	Adjusted OR (95% CI)	*p*-Value	Adjusted OR (95% CI)	*p*-Value	Adjusted OR (95% CI)	*p*-Value
Cholesterol						
Acute kidney injury	2.597 (0.794–8.493)	0.114	0.339 (0.090–1.283)	0.111	0.484 (0.101–2.317)	0.364
Respiratory failure	0.561 (0.143–2.196)	0.407	0.289 (0.016–5.362)	0.405	0.564 (0.022–14.40)	0.729
ICU	0.849 (0.215–3.350)	0.816	NA		NA	
Death	0.644 (0.156–2.667)	0.544	1.418 (0.058–34.90)	0.831	0.503 (0.046–5.465)	0.572
Triglycerides						
Acute kidney injury	1.179 (0.529–2.631)	0.687	0.779 (0.251–2.419)	0.666	1.666 (0.439–6.327)	0.453
Respiratory failure	2.293 (0.832–6.316)	0.108	0.364 (0.010–13.84)	0.587	0.330 (0.014–7.854)	0.493
ICU	1.714 (0.685–4.285)	0.249	NA		0.051 (0.001–1.764)	0.100
Death	1.180 (0.493–2.824)	0.711	NA		0.775 (0.215–2.793)	0.697

Odds ratios were calculated for total cholesterol at admission ≥200 mg/dL vs. <200 mg/dL and for triglyceride levels ≥150 mg/dL vs. <150 mg/dL. Parameters adjusted include age, sex, and pre-existing comorbidities (arterial hypertension, chronic obstructive pulmonary disease, chronic kidney disease, cerebrovascular disease, coronary heart disease, heart failure, and cancer). NA—non applicable.

**Table 7:** Association of CRP levels at admission with in-hospital complications by pneumonia etiology.

Complication and Outcome	COVID-19		Viral Infection		Bacterial Infection	
	Adjusted OR (95% CI)	*p*-Value	Adjusted OR (95% CI)	*p*-Value	Adjusted OR (95% CI)	*p*-Value
Acute kidney injury	0.999 (0.986–1.013)	0.932	0.995 (0.986–1.004)	0.262	1.011 (0.996–1.026)	0.144
Respiratory failure	1.015 (0.997–1.034)	0.095	1.020 (0.995–1.045)	0.119	1.027 (0.997–1.059)	0.081
ICU	1.016 (0.999–1.033)	0.061	NA		1.044 (0.989–1.102)	0.119
Death	1.015 (0.998–1.031)	0.081	1.003 (0.990–1.016)	0.682	1.039 (1.019–1.060)	<0.0001

CRP was included as a continuous variable in the logistic regression models. Parameters used for the adjustment include age, sex, and pre-existing comorbidities (arterial hypertension, chronic obstructive pulmonary disease, chronic kidney disease, cerebrovascular disease, coronary heart disease, heart failure, and cancer). NA—non applicable.

**Table 8:** Predictors of in-hospital complications by pneumonia etiology as dependent variables.

Complication and Outcome	COVID-19		Viral Infection		Bacterial Infection	
	Adjusted OR (95% CI)	*p*-Value	Adjusted OR (95% CI)	*p*-Value	Adjusted OR (95% CI)	*p*-Value
Acute kidney injury						
FPG at admission	0.801 (0.526; 1.219)	0.300	0.869 (0.469; 1.610)	0.656	0.813 (0.419; 1.580)	0.542
Glycemia variation	1.001 (0.997; 1.005)	0.571	1.001 (0.981; 1.022)	0.901	1.028 (1.011; 1.045)	0.001
CRP at admission	1.000 (0.989; 1.012)	0.935	0.998 (0.989; 1.006)	0.599	1.006 (0.992; 1.021)	0.389
Respiratory failure						
FPG at admission	0.792 (0.475; 1.320)	0.371	0.803 (0.308; 2.097)	0.654	1.989 (0.823; 4.809)	0.127
Glycemia variation	1.001 (0.996; 1.006)	0.660	0.992 (0.956; 1.030)	0.692	1.012 (0.995; 1.028)	0.169
CRP at admission	1.015 (1.000; 1.030)	0.052	1.000 (0.988; 1.011)	0.968	1.011 (0.990; 1.032)	0.297
ICU						
FPG at admission	1.519 (0.934; 2.472)	0.092	9.9 × 106 (0.000; -)	0.997	0.429 (0.119; 1.542)	0.195
Glycemia variation	1.004 (0.999; 1.009)	0.135	1.033 (0.923; 1.156)	0.577	1.004 (0.978; 1.030)	0.794
CRP at admission	1.015 (0.999; 1.031)	0.066	0.995 (0.950; 1.041)	0.819	1.022 (0.993; 1.051)	0.139
Death						
FPG at admission	1.750 (1.113; 2.754)	0.015	0.800 (0.259; 2.469)	0.698	0.673 (0.351; 1.288)	0.232
Glycemia variation	1.002 (0.998; 1.007)	0.309	0.957 (0.895; 1.023)	0.198	1.009 (0.994; 1.024)	0.236
CRP at admission	1.008 (0.994; 1.022)	0.243	1.006 (0.995; 1.017)	0.281	1.036 (1.018; 1.054)	<0.001

FPG—fasting plasma glucose; CRP—C-reactive protein. FPG at admission was included as <110 mg/dL (reference), 110–126 mg/dL, and ≥126 mg/dL. Glycemia variation and CRP were included in the analysis as continuous variables.

## Data Availability

The data presented in this study are available on request from the corresponding author.
